# Blood transfusion during cardiac surgery is associated with inflammation and coagulation in the lung: a case control study

**DOI:** 10.1186/cc10032

**Published:** 2011-02-11

**Authors:** Pieter R Tuinman, Alexander P Vlaar, Alexander D Cornet, Jorrit J Hofstra, Marcel Levi, Joost CM Meijers, Albertus Beishuizen, Marcus J Schultz, AB Johan Groeneveld, Nicole P Juffermans

**Affiliations:** 1 Department of Intensive Care Medicine and Laboratory of Experimental Intensive Care and Anesthesiology (LEICA), Academic Medical Center, Meibergdreef 9, Amsterdam, NL-1105 AZ, The Netherlands; 2 Department of Internal Medicine, Academic Medical Center, Meibergdreef 9, Amsterdam, NL-1105 AZ, The Netherlands; 3 Department of Experimental Vascular Medicine, Academic Medical Center, Meibergdreef 9, Amsterdam, NL-1105 AZ, The Netherlands; 4 Department of Intensive Care Medicine, VU University Medical Center, De Boelelaan 1117, Amsterdam, NL-1081 HZ, The Netherlands

## Abstract

**Introduction:**

Blood transfusion is associated with increased morbidity and mortality in cardiac surgery patients, but cause-and-effect relations remain unknown. We hypothesized that blood transfusion is associated with changes in pulmonary and systemic inflammation and coagulation occurring in patients who do not meet the clinical diagnosis of transfusion-related acute lung injury (TRALI).

**Methods:**

We performed a case control study in a mixed medical-surgical intensive care unit of a university hospital in the Netherlands. Cardiac surgery patients (*n *= 45) were grouped as follows: those who received no transfusion, those who received a restrictive transfusion (one two units of blood) or those who received multiple transfusions (at least five units of blood). Nondirected bronchoalveolar lavage fluid (BALF) and blood were obtained within 3 hours postoperatively. Normal distributed data were analyzed using analysis of variance and Dunnett's *post hoc *test. Nonparametric data were analyzed using the Kruskal-Wallis and Mann-Whitney *U *tests.

**Results:**

Restrictive transfusion increased BALF levels of interleukin (IL)-1β and D-dimer compared to nontransfused controls (*P *< 0.05 for all), and IL-1β levels were further enhanced by multiple transfusions (*P *< 0.01). BALF levels of IL-8, tumor necrosis factor α (TNFα) and thrombin-antithrombin complex (TATc) were increased after multiple transfusions (*P *< 0.01, *P *< 0.001 and *P *< 0.01, respectively) compared to nontransfused controls, but not after restrictive transfusions. Restrictive transfusions were associated with increased pulmonary levels of plasminogen activator inhibitor 1 compared to nontransfused controls with a further increase after multiple transfusions (*P *< 0.001). Concomitantly, levels of plasminogen activator activity (PAA%) were lower (*P *< 0.001), indicating impaired fibrinolysis. In the systemic compartment, transfusion was associated with a significant increase in levels of TNFα, TATc and PAA% (*P *< 0.05).

**Conclusions:**

Transfusion during cardiac surgery is associated with activation of inflammation and coagulation in the pulmonary compartment of patients who do not meet TRALI criteria, an effect that was partly dose-dependent, suggesting transfusion as a mediator of acute lung injury. These pulmonary changes were accompanied by systemic derangement of coagulation.

## Introduction

Blood transfusion can be a lifesaving intervention. However, it is increasingly recognized that transfusion itself contributes to morbidity and mortality in specific patient populations, including critically ill, cardiac surgery and trauma patients [1]. Transfusion-related acute lung injury (TRALI) is the most serious cause of transfusion-related morbidity and mortality [2,3] and is characterized by acute bilateral pulmonary permeability edema with subsequent hypoxia classically developing within 6 hours after transfusion [4].

Observational studies in critically ill patients indicate that transfusion is dose-dependently associated with acute lung injury (ALI) [5-8]. In these studies, however, the temporal relation between transfusion and adverse outcome has not clearly been determined. In an effort to capture the association between transfusion and ALI, the term "delayed TRALI" was coined [2], allowing ALI to develop after a longer time span than 6 hours. In line with this definition, TRALI criteria are fulfilled in only a minority of patients after cardiac surgery, although hypoxia is a frequent finding following this procedure [9-11]. Also, in a heterogeneous population of critically ill, transfusion of red blood cell units (RBCs) dose-dependently and transiently decreased oxygenation [12]. Together, this information may suggest that transfusion can result in lung injury without fulfilling the clinical consensus criteria of TRALI.

In contrast to this view, some authors argue that the association between blood transfusion and adverse outcome does not mean that transfusion actually mediates disease. It may merely be a marker of illness severity. Observational studies on the association of transfusion and adverse outcome have been recognized as sharing a common limitation: They do not distinguish between residual confounding, e.g. a sicker patient needing more transfusions, and actual causation [13-15].

To date, there are no clinical studies unequivocally showing the causal relationship between transfusion and ALI. Therefore, in the present study, we determined pulmonary and systemic effects of blood transfusion following cardiac surgery. We chose cardiac surgery patients for our study because cardiac surgery is a known risk factor for the development of TRALI [5] and because this group is a relatively homogeneous critically ill patient group who frequently undergo transfusion. We hypothesized that transfusion activates several pathways of inflammation that also mediate ALI and/or acute respiratory distress syndrome (ARDS) due to other causes and that such inflammatory processes may occur before patients meet the TRALI criteria. Pathways of interest include the production of proinflammatory cytokines [16] and chemotactic glycoproteins [17-19], as well as the activation of coagulation and the attenuation of fibrinolysis [20,21], all of which are found during lung injury [16,21]. Also, we determined whether the effects of transfusion accumulate with increased amounts of transfused blood, as dose dependency may be an additional indication of a causal relationship.

## Materials and methods

### Setting

The study was part of a larger trial performed in the mixed medical-surgical intensive care units (ICUs) of two university hospitals in the Netherlands [22]. The study in which 60 patients were included, was designed to look for an effect of transfusion on pulmonary permeability in cardiac surgery patients. Both ICUs are "closed format" departments in which patients are under the direct care of the ICU team. Patients included in the present analysis were chosen from among patients in one clinic, since samples for analysis were taken in only one clinic.

### Design

The study was approved by the Institutional Review Board (IRB 07/098# 07.17.0539). Prior to valvular and/or coronary artery bypass surgery, patients ages 18 years and older were asked for their informed consent for participation in the study. Exclusion criteria were off-pump surgery, emergency surgery or the use of immunosuppressive drugs. Patients were assigned to one of three groups: patients who received a restrictive transfusion of one or two red blood cells (RBCs) (*n *= 18); patients who underwent multiple transfusions, defined as transfusion of five or more units consisting of at least two RBCs, two fresh frozen plasma units and one unit of platelets of five donors (*n *= 10); and a control group receiving no transfusions (*n *= 17). The definition of multiple transfusions included transfusion of different blood products, which is a reflection of current transfusion practice. Transfusion was performed in the operation room or within the first 3 hours postoperatively. During the study, all transfused RBCs were leukoreduced (buffy coat removal, and the erythrocyte suspension was filtered to remove leukocytes (<1 × 10^6^), which is the standard of practice in the Netherlands) [23].

### Cardiothoracic surgery/anesthesia procedures

Patients were anesthetized according to the local institutional protocol with lorazepam, etomidate, sufentanil, and rocuronium for the induction of anesthesia, and with sevoflurane plus propofol for the maintenance of anesthesia. Steroids were given at the discretion of the cardioanesthesiologist. As part of standard care, a pulmonary artery catheter was inserted for perioperative monitoring. Cardiopulmonary bypass surgery was performed with the patient under mild to moderate hypothermia (28°C to 34°C) using a membrane oxygenator and a nonpulsatile blood flow. During the procedure, the patient's lungs were deflated. After the procedure, all patients were transferred to the ICU and placed on mechanical ventilation. Patients were ventilated in a pressure-controlled mode with tidal volumes targeted at 6 ml/kg.

### Nondirected bronchoalveolar lavage technique

Within 3 hours postoperatively, nondirected bronchoalveolar lavage was performed by instilling 20 ml of sterile 0.9% saline via a 50-cm, 14-gauge tracheal suction catheter as described previously [24,25]. In short, the distal end of the catheter was introduced via the endotracheal tube. Immediately after instillation of 20 ml of sterile 0.9% saline over 10 to 15 seconds, fluid was aspirated before withdrawal of the catheter.

### Specimen processing and assays

Bronchoalveolar fluid (BALF) and blood samples were centrifuged at 1,500 × *g *for 15 minutes, and the supernatant was stored at -80°C until assays were performed. Interleukin (IL)-1β, IL-4, IL-6, IL-8, tumor necrosis factor α (TNFα), von Willebrand factor (vWF), prothrombin fragments 1 and 2 (F1+F2), thrombin-antithrombin complexes (TATc) and plasminogen activator inhibitor type 1 (PAI-1) were measured using specific commercially available enzyme-linked immunosorbent (ELISAs) according to the instructions of the manufacturer (IL-1β, IL-4, IL-6, IL-8 and TNFα from PeliKine-compact™ kit, Sanquin, Amsterdam, the Netherlands; PAI-1, Hyphen BioMed, Andrésy, France; vWF antibodies, Dako, Glostrup, Denmark; F1+F2 and TATc, Siemens Healthcare Diagnostics, Marburg, Germany). D-dimer levels were determined with a particle-enhanced immunoturbidimetric assay (Innovance D-Dimer; Siemens Healthcare Diagnostics). Elastase-α_1_-antitrypsin complex (EA) levels [26] were measured by ELISA according to the instructions of the manufacturer (Sanquin).

Plasminogen activator activity (PAA%) was measured by an amidolytic assay [27]. Briefly, 25 μl of plasma were mixed to a final volume of 250 μl with 0.1 M Tris-Cl, pH 7.5, 0.1% (vol/vol) Tween 80, 0.3 mM S-2251 (Chromogenix, Mölndal, Sweden), 0.13 μM plasminogen, and 0.12 mg/ml cyanogen bromide fragments of fibrinogen (Chromogenix, Mölndal, Sweden). The results are expressed as percentages. Assays were performed batchwise to keep interassay variability as low as possible.

### Data collection

Preoperative European System for Cardiac Operative Risk Evaluation (EuroSCORE), the physical status classification system according to the American Society of Anesthesiologists (ASA score), predicted vital capacity, forced expiratory volume in 1 second and left ventricular function were determined. Left ventricular function was categorized as good (ejection fraction (EF) > 45%), moderate (EF < 45% but > 30%) or bad (EF ≤30%). Data on total operation room time, clamp time and time on heart-lung machine were extracted from the electronic patient data system. The duration of mechanical ventilation and the ratio of partial pressure of oxygen in arterial blood to inspired oxygen fraction (FiO_2_), or PaO_2_/FiO_2 _ratio, at the time of lavage were scored. Data on storage time of RBCs were obtained from the National Blood Bank. Suspected TRALI was scored using the consensus definition of ALI (new-onset hypoxemia or deterioration demonstrated by PaO_2_/FiO_2 _ratio < 300 mmHg within 6 hours after transfusion with bilateral pulmonary changes in the absence of cardiogenic pulmonary edema) [28-30]. Cardiogenic pulmonary edema was identified when pulmonary arterial occlusion pressure was > 18 mmHg or by the presence of at least two of the following: central venous pressure > 15 mmHg, preoperative a history of heart failure or valve dysfunction EF < 45% as estimated on the basis of an echocardiogram and a positive fluid balance. Chest radiographs were scored for the presence of new-onset bilateral interstitial abnormalities by two independent physicians who were blinded to the predictor variables. When interpretations differed, the chest radiograph and the description by the radiologist were reviewed to attain consensus.

### Statistics

Data were checked for distribution. Data are expressed as means (± SD) or medians (interquartile ranges) where appropriate. Boxplots display the lower hinge defined as the 25th percentile, middle as 50th percentile and upper hinge as the 75th percentile. Whiskers define lowest and highest observation. Normal distributed data were assessed using analysis of variance and Dunnett's *post hoc *test. Nonparametric data were analyzed using the Kruskal Wallis and Mann-Whitney *U *tests. A *P *value < 0.05 was considered statistically significant. Statistical analysis was performed using SPSS version 16.0 software (SPSS, Inc., Chicago, IL, USA).

## Results

Patient characteristics are shown in Table 1. The multitransfused group had a higher EuroSCORE compared to the other two groups. There were no differences in cardiac and pulmonary function or in clamp time between the groups. We found no difference in storage time of administered RBCs. The PaO_2_/FiO_2 _ratio after 3 hours on the ICU did not differ between multiple transfusion, restrictive transfusion and nontransfused patients (Table 1). There was also no difference in perioperative use of dexamethasone between groups. Multitransfused patients, however, received prolonged mechanical ventilation compared to restrictively transfused and nontransfused patients (Table 1). Of the transfused patients, only two met the clinical diagnosis of suspected TRALI.

**Table 1:** Demographics, baseline characteristics and perioperative data of cardiac surgery patients^a^

		Transfused		
			
Characteristics	Nontransfused (*n *= 17)	Restrictive (*n *= 18)	Multiple (*n *= 10)	*P *value
Age, years^b^	64 ± 11	64 ± 15	71 ± 6	0.231
Sex, male^c^	15 (88)	11 (61)	5 (50)	0.078
EuroSCORE^b^	3.8 ± 1.8	4.2 ± 2.4	8.5 ± 4.4	0.013
ASA score^b^	2.8 ± 0.6	3.0 ± 0.4	3.2 ± 0.4	0.067
Left ventricular function^c^				0.168
Bad	0 (0)	0	1 (10)	-
Moderate	6 (35)	4 (22)	4 (40)	-
Good	11 (65)	14 (78)	5 (50)	-
FEV_1_, percentage of predicted value^d^	91 (24)	98 (25)	84 (23)	0.281
Type of surgery^c^				0.763
CABG	12 (71)	9 (50)	5 (50)	-
Valve replacement	2 (12)	6 (33)	1 (10)	-
Other	3 (18)	3 (18)	4 (40)	-
Clamp time, minutes^d^	55 (47)	67 (54)	79 (62)	0.151
Pump time, minutes^d^	99 (60)	90 (68)	107 (90)	0.181
OR time, minutes^d^	313 (126)	315 (95)	339 (156)	0.201
CVP, mmHg^b^	7.7 (5.9)	7.7 (5.6)	7.8 (9.6)	0.855
CO, l/min^d^	4.7 (1.7)	3.8 (2.9)	4.4 (2.2)	0.269
Storage time RBCs, days^d^	-	14.5 (10)	15.0 (8)	0.481
PaO_2_/FiO_2 _ratio^b^	305 ± 153	343 ± 94	289 ± 106	0.458
Hb at ICU, mM/L^b^	5.7 ± 0.7	5.6 ± 0.7	5.0 ± 0.4	0.055
aPTT at ICU, seconds^d^	26 (4)	27 (3)	34 (7)	0.001
PTT at ICU, seconds^d^	12 (0.5)	12 (1.3)	18 (2.1)	0.001
MV, total time on ICU, hours^d^	10 (8)	14 (9)	19 (6)	0.009

^a^EuroSCORE, European System for Cardiac Operative Risk Evaluation; ASA score, physical status classification system according to the American Society of Anesthesiologists; FEV_1_, forced expiratory volume in 1 second, given as percentage of predicted value; CABG, coronary artery bypass graft surgery; CVP, central venous pressure; CO, cardiac output; PaO_2_/FiO_2_, ratio of partial pressure of oxygen in arterial blood (PaO_2_) to inspired oxygen fraction (FiO_2_); ICU, intensive care unit; OR, operation room time; RBCs, red blood cells; Hb, hemoglobin; aPTT, activated partial thromboplastin time; PTT, partial thromboplastin time; MV, mechanical ventilation; ^b^mean ± SD; ^c^counts (%); ^d^median (IQR).

### Effect of blood transfusion on pulmonary and systemic inflammation

**Figure 1:**
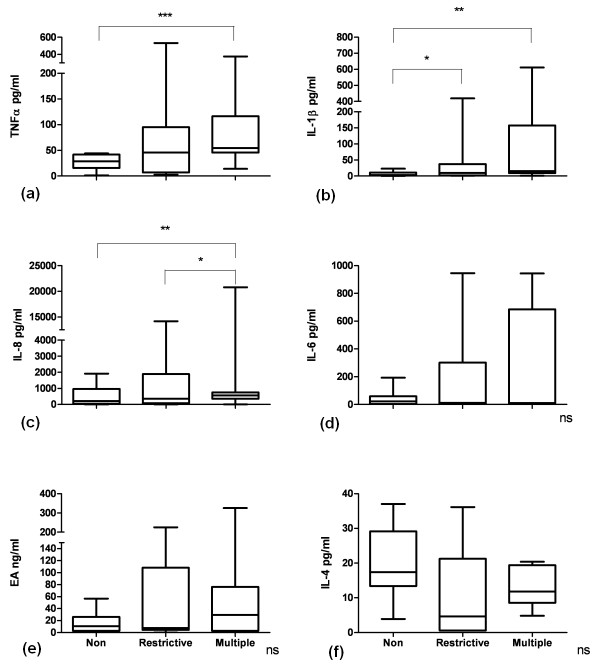
**Boxplots showing cytokine levels in the bronchoalveolar fluid of cardiac surgery patients**. **(a) **TNFα, tumor necrosis factor α. ***P < 0.001. **(b) **Interleukin (IL)-1β. * *P *< 0.05; ** *P *< 0.01. (c) * *P *< 0.05; ** *P *< 0.01. **(d) **ns, not significant. **(e) **EA, elastase-α_1_-antitrypsin complex; Non, nontransfused (*n *= 17); Restrictive, 1 or 2 units of blood transfused (*n *= 18); Multiple, ≥ 5 units of blood transfused (*n *= 10). **(f) **ns, not significant. Nonparametric tests were used for analysis. Boxplots: the lower hinge defined as the 25th percentile, middle as 50th percentile and upper hinge as the 75th percentile. Whiskers define lowest and highest observation.

Transfusion was associated with an increase in levels of TNFα, IL-1β and IL-8 in BALF compared with nontransfused patients (Figure 1). Multiply transfused patients had higher levels of IL-8 compared to restrictively transfused patients. Transfusion tended to increase pulmonary IL-6 and EA levels and to decrease IL-4 levels compared to nontransfused controls (Figure 1).

In the systemic compartment, multiple transfusions were associated with an increase in TNFα compared to restrictively transfused and nontransfused patients (data presented as median (IQR): 312 pg/ml (345) versus 64 pg/ml (127) versus 182 pg/ml (190), respectively; *P *< 0.01). EA levels in plasma were nonsignificantly elevated after multiple and restrictive transfusion compared to nontransfused controls (287 (441) versus 256 (254) versus 202 (249) ng/ml respectively, *P *= 0.50) (data not shown in a graph). Other markers of systemic inflammation, including plasma levels of Il-1β, IL-4, IL-6 and IL-8 were not clearly affected by blood transfusion (data not shown).

The BALF/plasma ratios of transfused patients for IL-1β, IL-4 and IL-8 were evidently greater than 1 (141, 10 and 375, respectively), indicating that inflammation is more pronounced in the pulmonary compartment. For the other cytokine levels, the mean ratio had a value of around 1, indicating that the level of the cytokines in the pulmonary compartment equaled the level in the systemic compartment after transfusion. In the nontransfused group, the BALF/plasma ratios for IL-1β, IL-4 and IL-8 were greater than 1 (6, 12 and 12, respectively), whereas the ratios for IL-6 and TNFα were clearly below 1 (0.22 and 0.15, respectively).

### Effect of blood transfusion on pulmonary and systemic coagulation and fibrinolysis

**Figure 2:**
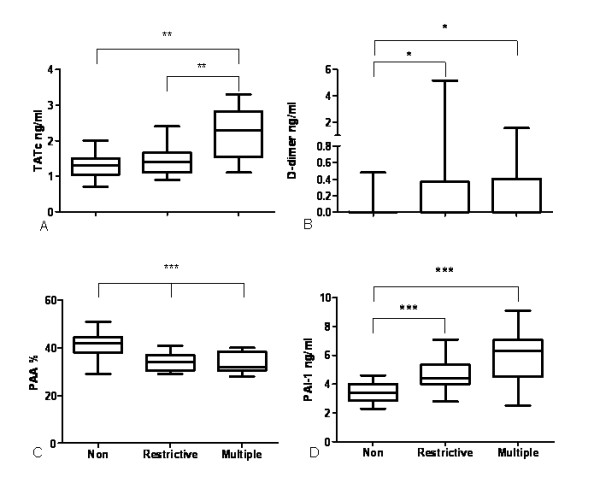
**Boxplots showing markers of coagulation and fibrinolysis in the bronchoalveolar fluid of cardiac surgery patients**. **(a) **TATc, thrombin-antithrombin complexes. **P < 0.01. (b) *P < 0.05. ***P < 0.001, **P < 0.01, *P < 0.05. **(c) **PAA %, plasminogen activator activity percentage; Non, nontransfused (*n *= 17); Restrictive, 1 or 2 units of blood transfused (*n *= 18); Multiple, ≥ 5 units of blood transfused (*n *= 10). ***P < 0.001. **(d) **PAI-1, plasminogen activator inhibitor type 1. ***P < 0.001. Nonparametric tests were used for analysis for TATc and PAI-1, and a parametric test was used for analysis of PAA %. Boxplots: the lower hinge defined as the 25th percentile, middle as 50th percentile and upper hinge as the 75th percentile. Whiskers define lowest and highest observation.

Multiple blood transfusions were associated with activation of pulmonary coagulation, exemplified by an increase in BALF levels of TATc compared to restrictively transfused and nontransfused controls (Figure 2). For D-dimer, we found higher levels after both restrictive and multiple transfusions compared to nontransfused controls (Figure 2). BALF levels of PAA% were lower in multiply transfused patients compared to restrictively transfused patients and nontransfused controls, indicative of impaired fibrinolysis. The decrease in PAA% may have been due to an increase in BALF levels of PAI-1 in transfused patients compared to nontransfused patients (Figure 2). Levels of vWF and F1+F2 were not significantly different between groups (data not shown).

**Figure 3:**
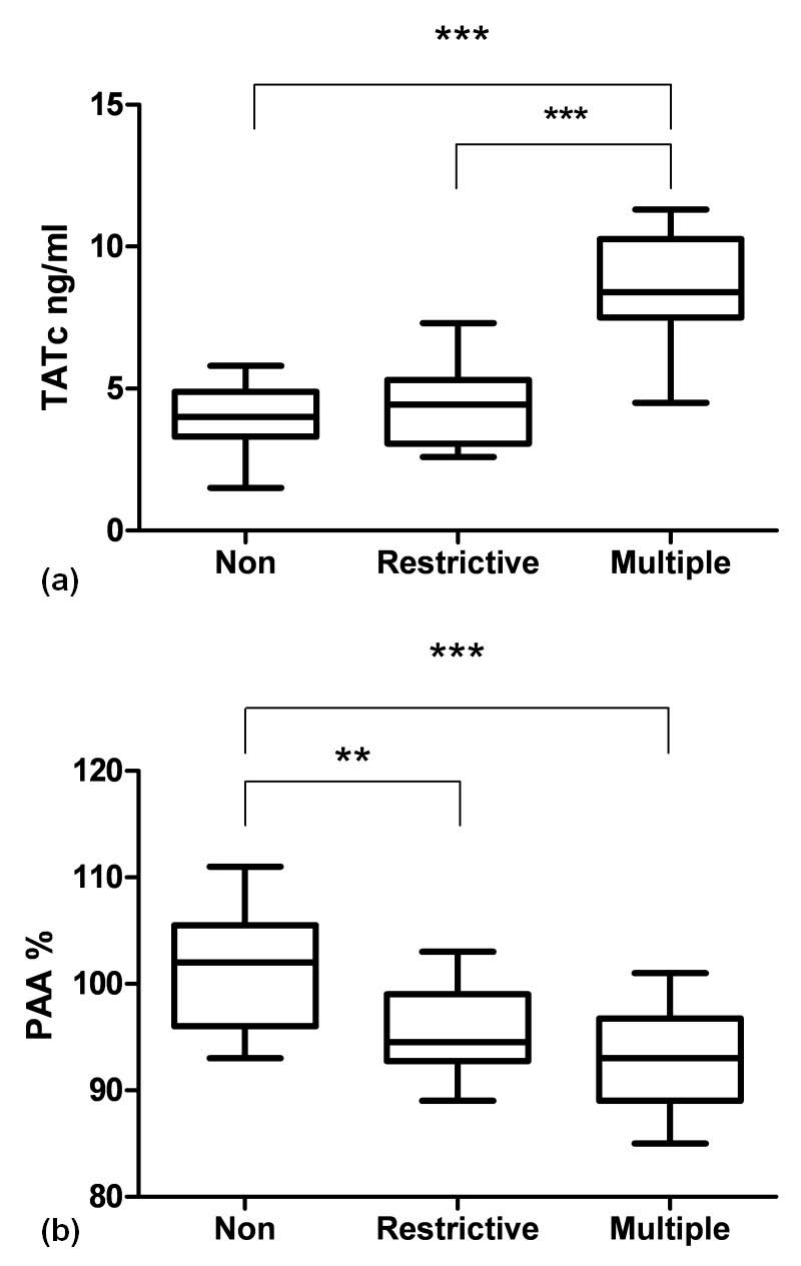
**Boxplots showing plasma levels in cardiac surgery patients**. **(a) **thrombin-antithrombin complexes (TATc). ***P < 0.001 (nonparametric test). **(b) **plasminogen activator activity percentage (PAA %). **P < 0.01; ***P < 0.001 (parametric tests). Non, nontransfused (*n *= 17); Restrictive, 1 or 2 units of blood transfused (*n *= 18); Multiple, ≥ 5 units of blood transfused (*n *= 10). Boxplots: the lower hinge defined as the 25th percentile, middle as 50th percentile and upper hinge as the 75th percentile. Whiskers define lowest and highest observation.

Transfusion had a clear effect on markers of coagulation in the systemic compartment. In plasma, we found a significantly higher level of TATc in transfused patients compared to nontransfused controls (Figure 3). Also, fibrinolysis was attenuated as indicated by a decrease in the level of PAA% in transfused patients compared to nontransfused controls (Figure 3). Levels of D-dimer, vWF and F1+F2 were not significantly different between groups (data not shown).

**Figure 4:**
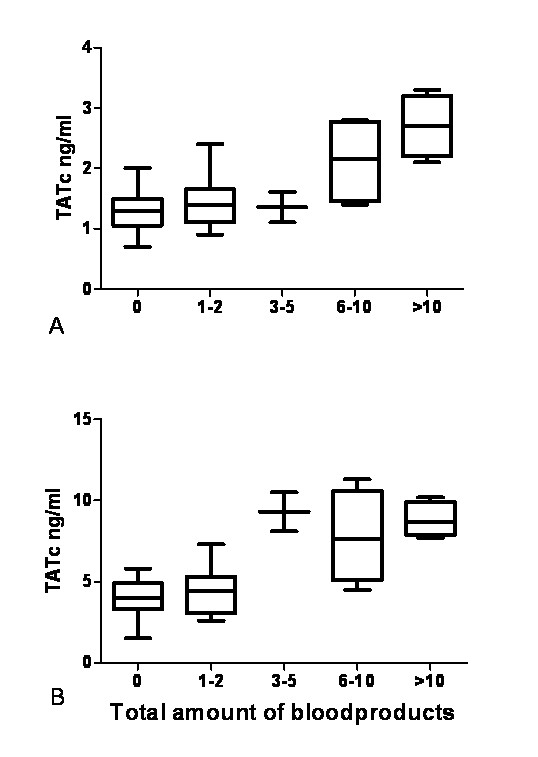
**Boxplots showing TATc according to amount of blood products transfused per patient**. Boxplots showing thrombin-antithrombin complexes (TATc) in **(a) **bronchoalveolar fluid and **(b) **plasma according to the total amount of blood products given per patient. Boxplots: the lower hinge defined as the 25th percentile, middle as 50th percentile and upper hinge as the 75th percentile. Whiskers define lowest and highest observation.

The response to transfusion was clearly dose-dependent for TATc in BALF and TATc in plasma as shown in Figure 4 (Pearson's correlation coefficient r = 0.694 and *P *< 0.001, and Pearson's correlation coefficient r = 0.730, *P *< 0.001, respectively), but was also apparent for TNFα and PAA% in plasma and for IL-1β, PAA% and PAI-1 in BALF (data not shown).

The BALF/plasma ratios of transfused patients for D-dimer, TATc and PAA% were evidently smaller than 1 (0.16, 0.28 and 0.36, respectively), and in nontransfused controls the BALF/plasma ratios were also smaller than 1 (0.01, 0.42 and 0.40, respectively), indicating that activation of coagulation and impaired fibrinolysis were more pronounced in the systemic compartment.

Multiply transfused patients had a higher risk of complications following surgery compared to restrictively transfused and nontransfused patients, exemplified by a higher EuroSCORE. The EuroSCORE is calculated using age as well as pulmonary and myocardial function. To account for confounding patient-related effects, we stratified patients according to their EuroSCORE as low (0 to 2, *n *= 8), moderate (3 to 5, *n *= 19) or high (≥ 6, *n *= 16) risk [31] and reanalyzed the data according to these groups. We found no difference in the BALF levels of markers of inflammation and coagulopathy between groups or with regard to plasma levels (data not shown). Also, duration of mechanical ventilation (data presented as median (IQR): 14 (8) versus 12 (12) versus 14 (15) hours, respectively, *P *= 0.368) was not different between patients when stratified according to EuroSCORE.

## Discussion

In this study, blood transfusion during cardiac surgery was associated with a marked pulmonary inflammatory reaction, partly in a dose-dependent manner, and was characterized by enhanced levels of proinflammatory cytokines and bronchoalveolar activation of coagulation and inhibition of fibrinolysis. Transfusion also was associated with systemic activation of coagulation, impaired fibrinolysis and, to a lesser extent, with systemic inflammation. Furthermore, we confirm that the amount of transfusion was associated with longer mechanical ventilation in the ICU.

The finding that blood transfusion is associated with inflammation and activation of coagulation and impaired fibrinolysis in the lungs may indicate a mechanism of the observed association between transfusion and postoperative morbidity in cardiac surgery patients [8]. Transfusion has previously been shown to upregulate inflammatory genes and cytokine production [32-34]. To our knowledge, data on pulmonary effects are limited. In this study, the pulmonary inflammatory response after transfusion was characterized by an elevation of IL-1β, IL-8 and TNFα. In accordance, packed RBCs were found to stimulate leukocyte IL-8 gene expression *in vitro *and to activate neutrophils to release IL-8 [32,35]. Also, donor plasma was shown to activate peripheral mononuclear cells to produce a wide array of inflammatory mediators, including IL-1β, IL-6, TNFα and IL-8, *in vitro *[34]. Furthermore, there was a trend toward higher levels of IL-6 and EA and lower levels of the anti-inflammatory cytokine IL-4 after transfusion. These same cytokines are known to be involved in ALI and ARDS [16]. Concurrently, BALF levels of IL-6 and IL-8 are correlated with the development of ARDS [36], and high BALF levels of TNFα, IL-1, IL-6 and IL-8 are associated with increased mortality [37]. Inflammation and coagulation have tight interaction; that is, they stimulate each other in both proinflammatory and procoagulant directions [36,38].

We found that blood transfusion is associated with activation of pulmonary coagulation and impairment of fibrinolysis. Coagulopathy is a distinct feature of ALI and ARDS due to other causes [16,20,21], contributing to morbidity and mortality [39]. In animals, massive transfusion resulted in extensive numbers of microemboli in the pulmonary vasculature [40]. As the endothelium initiates and regulates coagulation [41], it can be hypothesized that coagulopathy may also play a role in ALI following the systemic "hit" of a blood transfusion. In accordance, we recently showed that lung injury following transfusion was characterized by profound pulmonary and systemic coagulopathy in a two-hit murine transfusion model [42,43]. Also in this study, transfusion was associated with clear systemic activation of coagulation, whereas systemic inflammation was only mild. A possible mechanism of the observed coagulation derangements may be activation of coagulation factor IX by the membranes of erythrocytes, which in turn is capable of activating factor X, leading to thrombin generation [44]. Of interest is the finding that transfusion was dose-dependently associated with an increase in the levels of PAI-1, since an increase in PAI-1 levels is of prognostic significance in patients with ALI and/or ARDS [39], sepsis [45] and pneumonia [46]. Therefore, it may be a marker of pulmonary complications.

The observed effects of transfusion were dose-dependent, at least partially. In agreement with this effect, observational studies have shown that the number of erythrocytes transfused is associated with the onset of TRALI as well as with adverse outcome [7,47]. However, these observational data cannot distinguish confounding effects from causation [13,15]. The finding of a dose-dependent relationship for the observed inflammatory reaction may contribute to the suggestion that transfusion is a mediator of lung injury and not merely a marker. Of note, not all parameters were dose-dependently affected. However, given that markers showed the same trend, we propose that this may be due to the small sample size.

The findings that a single transfusion already elicits pulmonary inflammation and that these alterations are dose-dependent support a restrictive transfusion strategy. However, blood transfusion cannot be avoided altogether, in particular not in cardiac surgery patients, calling for other strategies to limit pulmonary complications following transfusion. In cardiac surgery patients, an association between nonleukoreduced blood transfusion and mortality was found [48]. Although leukoreduction reduces levels of cytokines in stored blood, adverse transfusion-related outcomes continue to occur [49]. In line with these data, we have shown that leukoreduced blood enhances inflammation and coagulation in the lung in cardiac surgery patients. Thus, leukoreduction may not protect against the occurrence of ALI. Storage time has been implicated in increased risk of postoperative complications as well as reduced short-term and long-term survival in patients undergoing cardiac surgery [3]. Since we found no difference in RBC storage time between restrictively and multiply transfused patients, storage time did not account for the observed differences between the groups.

This study has several limitations. Multiply transfused patients had a higher EuroSCORE than restrictively transfused and nontransfused patients and displayed a trend for a longer time on the cardiopulmonary bypass machine. Therefore, EuroSCORE and duration of cardiopulmonary bypass may have contributed to the proinflammatory response and derangement of coagulation. Therefore, we performed a separate analysis stratifying groups according to low, moderate and high EuroSCORE. We found no differences in levels of inflammatory cytokines and markers of coagulopathy between the three groups. These results suggest that the observed effects might be attributable to blood transfusion. In line with this hypothesis, some effects of transfusion were apparent already after restrictive transfusion, and this patient group did not differ in EuroSCORE and time on heart-lung machine compared to nontransfused controls. In accordance, in a previous study showing increased cytokine levels in transfused cardiac surgery patients, it was shown that transfusion, and not cardiopulmonary bypass, was the most important source for the inflammatory response [33]. In addition, in a prospective study of the mechanisms of TRALI in cardiac surgery patients, we recently found that cardiopulmonary bypass resulted in transient inflammation which subsided at the time of onset of TRALI [50]. Taken together, these results may be compatible with the suggestion that blood transfusion mediates pulmonary inflammation. However, we cannot exclude that other confounding factors unaccounted for, such as pump time, may have played a role in the observed inflammation and activated coagulation. Furthermore, our data cannot be applied to a general ICU population, since we studied only cardiac surgery patients. A final limitation of this study is the use of multiple comparisons, which can yield a significant difference that actually relies on chance. However, for the majority of differences found in this study, the *P *value was below 0.01.

## Conclusions

We have shown that transfusion is associated with pulmonary and systemic inflammation as well as with activation of coagulation and impaired fibrinolysis, an effect that was in part dose-dependent. These data may indicate that transfusion is a mediator of lung inflammation in patients after cardiac surgery and not merely a marker of disease. Insight into the effects of blood transfusion may contribute to the risk-benefit assessment of the decision to initiate blood transfusion in cardiac surgery patients.

## Key messages

• Blood transfusion during cardiac surgery is associated with marked pulmonary inflammatory reaction, partly in a dose-dependent manner.

• This inflammation is characterized by bronchoalveolar activation of coagulation and inhibition of fibrinolysis.

• Transfusion was also associated with systemic derangement of coagulation and, to a lesser extent, systemic inflammation.

• The amount of transfusion is associated with longer mechanical ventilation on the ICU.

• These data indicate that transfusion may be a mediator of lung injury in cardiac surgery patients.

## Abbreviations

ALI: acute lung injury; ARDS: acute respiratory distress syndrome; ASA: score, physical status classification system according to the American Society of Anesthesiologists; BALF: bronchoalveolar lavage fluid; EA: elastase-α_1_-antitrypsin complex; EuroSCORE, preoperative European System for Cardiac Operative Risk Evaluation; FEV_1: _forced expiratory volume in 1 second; ICU: intensive care unit; IL: interleukin; IQR: interquartile range; OR, total operation room time; PAA%: plasminogen activator activity level; PAI-1: plasminogen activator inhibitor type 1; RBC: red blood cell unit; SD: standard deviation; SEM: standard error of the mean; TATc: thrombin-antithrombin complex; TNFα: tumor necrosis factor α; TRALI: transfusion-related acute lung injury; vWF: von Willebrand factor.

## Competing interests

The authors declare that they have no competing interests.

## Authors' contributions

PRT was intimately involved in interpretation of the results as well as in manuscript preparation. He was also involved in data extraction as well as statistics. He read the final version of the manuscript and agrees with all reported findings and interpretations. APV was instrumental in the coordination of the study and the performance of data gathering. He carried out the cytokine ELISAs. He was also intimately involved with interpretations of the results. He read the final version of the manuscript and agrees with all reported findings and interpretations. ADC was instrumental in the coordination of the study, data gathering and analysis. He read the final version of the manuscript and agrees with all reported findings and interpretations. JJH was instrumental in data gathering and analysis. He read the final version of the manuscript and agrees with all reported findings and interpretations. ML was instrumental in performing the coagulation and fibrinolysis assays and helped to draft the manuscript. He read the final version of the manuscript and agrees with all reported findings and interpretations. JCMM was instrumental in data analysis and extraction. He read the final version of the manuscript and agrees with all reported findings and interpretations. AB was instrumental in the study's hypothesis and design. He read the final version of the manuscript and agrees with all reported findings and interpretations. MJS was instrumental in the study's hypothesis and design. He read the final version of the manuscript and agrees with all reported findings and interpretations. ABJG was instrumental in the study's hypothesis and design. He read the final version of the manuscript and agrees with all reported findings and interpretations. NPJ was instrumental in developing the study's hypothesis and was intimately involved in the interpretation of the results as well as in manuscript preparation and data statistics. She read the final version of the manuscript and agrees with all reported findings and interpretations.
